# Interactions between Lipopolysaccharide and Peptide Bacteriocin BacSp222 Influence Their Biological Activities

**DOI:** 10.1021/acsinfecdis.5c00066

**Published:** 2025-07-08

**Authors:** Justyna Śmiałek-Bartyzel, Monika Bzowska, Alicja Frączek, Iwona Wojda, Renata Mężyk-Kopeć, Piotr Bonarek, Artur Blat, Jan Rak, Paweł Mak

**Affiliations:** † Doctoral School of Exact and Natural Sciences, 37799 Jagiellonian University, Łojasiewicza 11 Street, Kraków 30-348, Poland; ‡ Department of Analytical Biochemistry, Faculty of Biochemistry, Biophysics and Biotechnology, Jagiellonian University, Gronostajowa 7 Street, Kraków 30-387, Poland; § Department of Cell Biochemistry, Faculty of Biochemistry, Biophysics and Biotechnology, Jagiellonian University, Gronostajowa 7 Street, Kraków 30-387, Poland; ∥ Department of Immunobiology, Institute of Biological Sciences, Maria Curie-Skłodowska University, Akademicka 19, Lublin 20-033, Poland; ⊥ Department of Physical Biochemistry, Faculty of Biochemistry, Biophysics and Biotechnology, Jagiellonian University, Gronostajowa 7 Street, Kraków 30-387, Poland; # Małopolska Centre of Biotechnology, Jagiellonian University, Gronostajowa 7A Street, Kraków 30-387, Poland

**Keywords:** bacteriocin, BacSp222, inflammation, lipopolysaccharide/LPS, tumor necrosis factor/TNF, toll-like receptors/TLR

## Abstract

This study describes the interactions between two different pro-inflammatory factors produced by bacteria, lipopolysaccharide (LPS) from Gram-negative bacteria and the peptide BacSp222 produced by a Gram-positive zoonotic strain, 222. We demonstrate that the mentioned molecules interact, forming a complex, and this phenomenon selectively reduces their biological activities in vitro and in vivo. Specifically, the levels of tumor necrosis factor (TNF) and nitric oxide (NO) produced by monocyte-macrophage cells were lower in samples treated with both LPS and BacSp222 compared to those treated with LPS alone. This is most likely because BacSp222 limited the ability of LPS to stimulate the TLR4 receptor. In the larvae injected simultaneously with LPS and BacSp222, the activity of hemolymph phenoloxidase, a key component of the insect immune response, was lower than that observed in larvae injected with either LPS or BacSp222 alone. Moreover, LPS inhibited the antibacterial activity of the bacteriocin, while BacSp222 limited LPS’s ability to activate a proenzyme in the amebocyte lysate test. The changes in the activities of BacSp222 and LPS were attributed to the electrostatic interactions between LPS micelles and bacteriocin molecules, resulting in a decrease in LPS aggregate size and the direct formation of a complex between them, as revealed by gel filtration and isothermal microcalorimetry.

## Graphical abstract

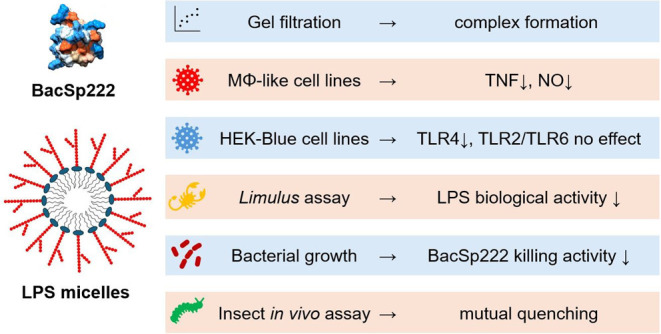

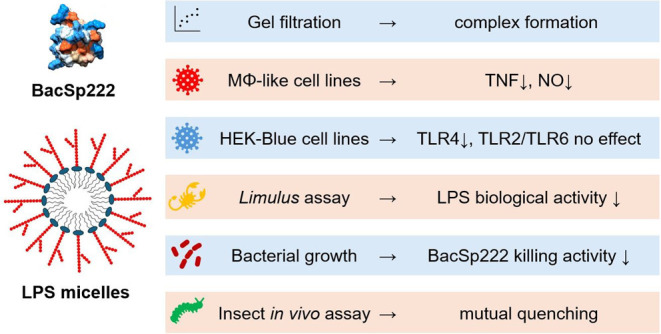

Bacteriocins play a key role in the biology, ecology, and evolution of bacteria, allowing them to compete for resources and space in the environment. These molecules are proteins or peptides produced by bacteria ribosomally and secreted into the surrounding microenvironment, where they act as antibacterial factors, especially against closely related species. In the case of opportunistic or pathogenic bacteria, the production of bacteriocins gives a significant competitive advantage to the producer’s cells, especially in microbially diverse environments, such as the skin, mucous membranes, or gastrointestinal tract of higher organisms. Bacteriocins are then tools of competitive warfare, allowing their producers to eliminate other microorganisms that could limit their growth or access to nutrients in host organisms.^1–3^ However, more and more reports are expanding the biological role of bacteriocins beyond the direct regulation of microbiota composition and proving that these molecules possess immunomodulatory activity.^4–6^

A good example of such a multifunctional and immunomodulatory bacteriocin is the peptide BacSp222, produced by an opportunistic canine pathogen, strain 222.^7^ BacSp222 is a 50 amino acid long linear peptide encoded on a plasmid and is produced without a leader sequence, retaining a formylated N-terminal methionine. Its amino acid sequence seems to be unique, but the general physicochemical properties of BacSp222 resemble features of a group of several previously described bacteriocins of, predominantly, different staphylococci: lacticins Q and Z, aureocins A53 and AurK411, capidermicin, and epidermicin NI01.^8–12^ The BacSp222 molecule forms a bundle of four helices and kills a wide range of Gram-positive bacteria by disrupting their cellular membranes through a barrel-stave pore formation mechanism.^13,14^ However, in addition to such a primary function, BacSp222 can also modulate the activity of the immune system. Our recent studies demonstrated that the peptide is recognized by the TLR2/TLR6 receptor heterodimer and, through myeloid differentiation primary response 88 protein (MyD88)-dependent activation of nuclear factor kappa-light-chain-enhancer of activated B cells (NF-κB) transcription factor, induces the synthesis of many pro-inflammatory cytokines, including tumor necrosis factor (TNF), monocyte chemoattractant protein-1 (MCP-1), interleukin-1 alpha (IL-1-alpha), and interleukin-8 (IL-8). Furthermore, in the presence of interferon-gamma (IFN-gamma), the peptide is capable of stimulating mouse immune cells to express an inducible nitric oxide synthase and, consequently, to secrete nitric oxide (NO), a key microbicidal effector and signaling molecule during inflammation.^15,16^

However, unlike typical pro-inflammatory molecules, BacSp222 cannot activate human neutrophils to produce reactive oxygen species (ROS) and induce the production of extracellular neutrophil traps (NETs). Furthermore, contrary to other formylated bacterial peptides, BacSp222 cannot activate a separate group of pathogen-detecting and immunomodulatory receptors, such as the formyl peptide receptors FPR1 and FPR2.^15^ Although kinetic studies revealed that BacSp222 has a lower affinity for the TLR2/TLR6 heterodimer than synthetic ligands based on lipoteichoic acids or lipopeptides,^10^ BacSp222 is the first known representative of bacteriocins for which the direct ability to activate Toll-like receptors has been demonstrated.

The present study sheds more light on the multiple biological activities of BacSp222 and further makes the image of this peptide even more complex. As stated above, BacSp222 kills a broad range of Gram-positive bacteria, but on the other hand, it is inactive against Gram-negative ones. The presented study revealed that preincubation of the BacSp222 molecule with lipopolysaccharide (LPS), the most important endotoxin of Gram-negative bacteria, decreased or eliminated the biological activities of both of the mentioned molecules. The present study shows the mutual affinity of BacSp222 and LPS as well as the physiological consequences of this phenomenon, especially in the context of the key role of LPS in the induction of the inflammatory response.

## Results

### BacSp22 Modulates LPS-Induced TNF Production by Murine Monocytic-Macrophage Cells

The interaction of various bacteriocins with LPS has been previously reported, but the biological relevance of such a phenomenon is diversified.^17–19^ The present research investigated the impact of the staphylococcal bacteriocin BacSp222 on the inflammatory response of cells treated with LPS. Two LPS preparations were used in the experiments from serotypes O55:B5 and O111:B4. Both are commonly applied in biomedical studies^20,21^ and belong to the R3 type of lipopolysaccharides, containing all three typical chemical structures (i.e., O-specific polysaccharides, core oligosaccharides, and lipid A) but differ in details of composition of O-antigen, and, to a lesser degree, core oligosaccharides. We used the highest available purity commercial LPS preparations, obtained by ion-exchange chromatography—O55:B5 (IEC) and O111:B4 (IEC). Additionally, to verify the possible effect of the degree of purity on biological activity, we used in some experiments a lower-quality formulation of O111:B4 LPS obtained by phenol extraction, O111:B4 (PE). The commercial details concerning the mentioned preparations of LPS are given in the Methods section.

We simultaneously stimulated murine monocyte-macrophage cell lines with both LPS and BacSp222 and then measured the TNF levels in the postculture media. Our results showed that the cells treated simultaneously with LPS and BacSp222 produced less TNF than those treated only with LPS (Figure 1). This decrease in cytokine levels was observed across all LPS preparations used for each of the tested cell lines. However, the differences were not statistically significant for RAW 264.7 cells treated with LPS O111:B4 (IEC) and P388.D1 cells treated with LPS O55:B5 (IEC). The most significant differences in TNF levels between the cells treated by LPS only and those treated with both LPS and BacSp222 were noted for the LPS O111:B4 (PE) preparation.

**Figure 1:**
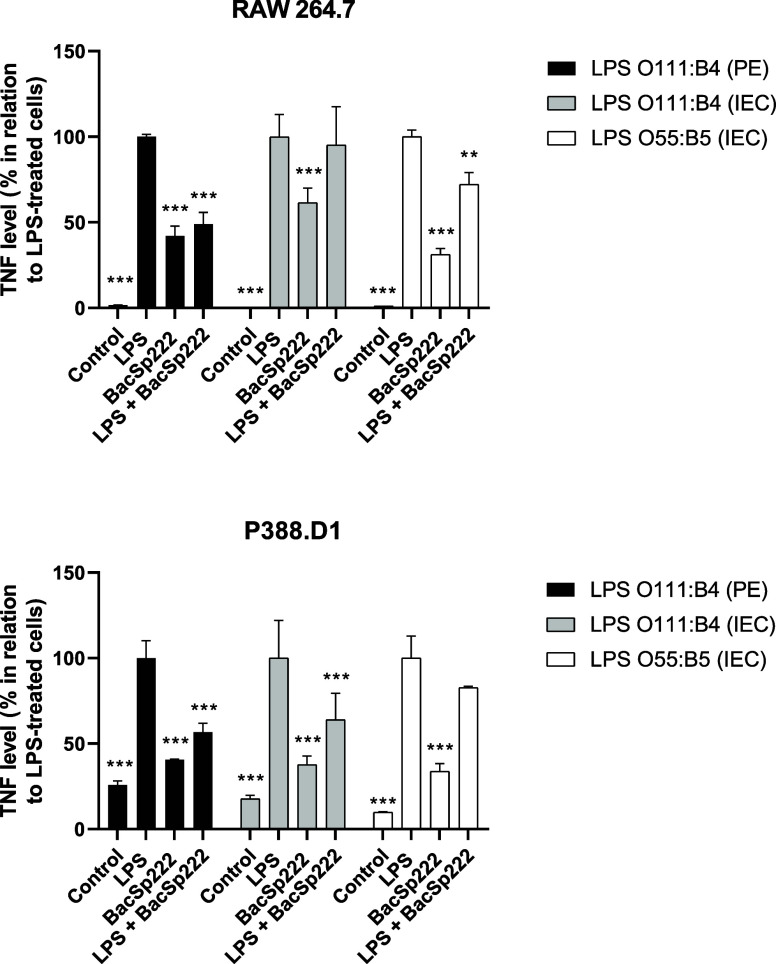
Analysis of the level of TNF produced by murine monocyte/macrophage cell lines treated simultaneously with BacSp222 and LPS. The cells were cultured for 6 h in a medium (control) or a medium containing various types of LPS (100 ng/mL), BacSp222 (1 μM), or LPS with BacSp222. After stimulation, the level of TNF in postculture media was determined. The bars represent the mean ± SD (*n* = 3), *p < 0.05, **p < 0.01, ***p < 0.001 vs LPS-treated cells.

Knowing that the presence of bacteriocin reduces the amount of TNF produced by LPS-stimulated cells, we investigated whether simultaneous costimulation with BacSp222 and LPS would affect the level of NO released by murine monocyte–macrophage cells. For this purpose, we treated the cells for 24 h with either LPS alone or LPS combined with BacSp222 and then measured the level of NO released into the medium. In the case of RAW 264.7 cells, the simultaneous costimulation with bacteriocin and LPS resulted in a reduced level of NO release compared with stimulation with endotoxin alone. Statistically significant differences were observed for two LPS preparations, O111:B4 (PE) and O55:B5 (IEC) (Figure 2). However, for P388.D1 cells, a statistically significant reduction in NO levels in the postculture media was noted only for the LPS preparation of the O111:B4 (IEC) when treated simultaneously with the peptide and LPS (Figure 2).

**Figure 2:**
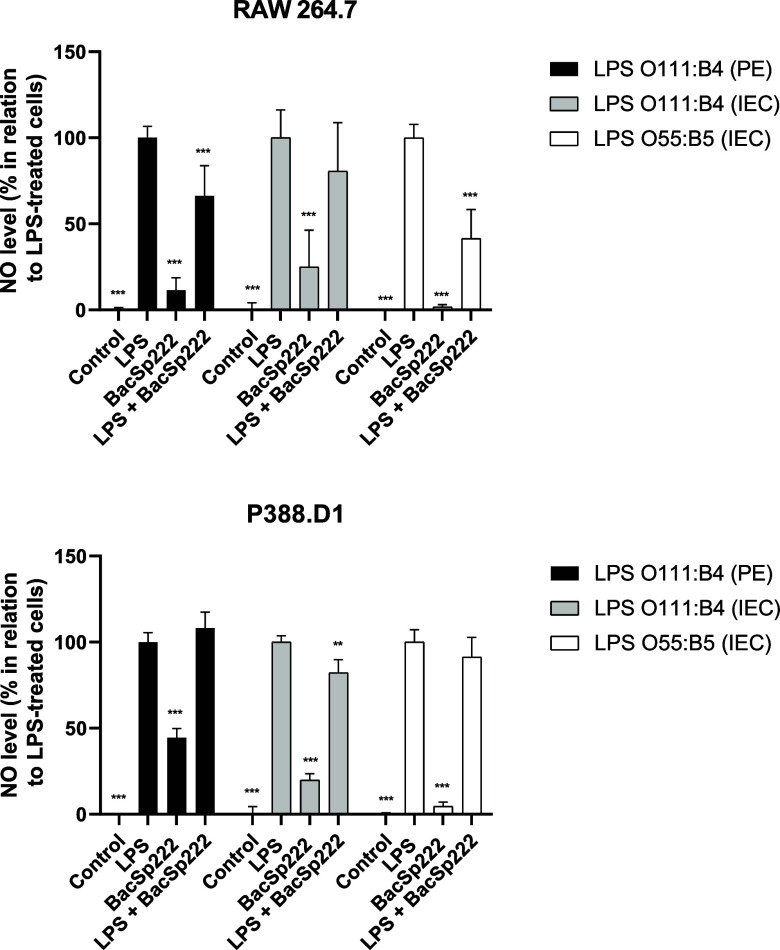
Analysis of NO production by murine monocyte/macrophage cell lines treated simultaneously with BacSp222 and LPS. The cells were cultured for 24 h in a medium (control) or a medium containing various types of LPS (100 ng/mL), BacSp222 (1 μM), or LPS with BacSp222. After stimulation, the level of NO in postculture media was measured. The bars represent the mean ± SD (*n* = 3), *p < 0.05, **p < 0.01, ***p < 0.001 vs LPS-treated cells.

Additionally, we confirmed that 24 h stimulation of RAW 264.7 and P388.D1 cells with LPS and BacSp222 simultaneously did not affect their viability compared to unstimulated cells (Figure 3).

**Figure 3:**
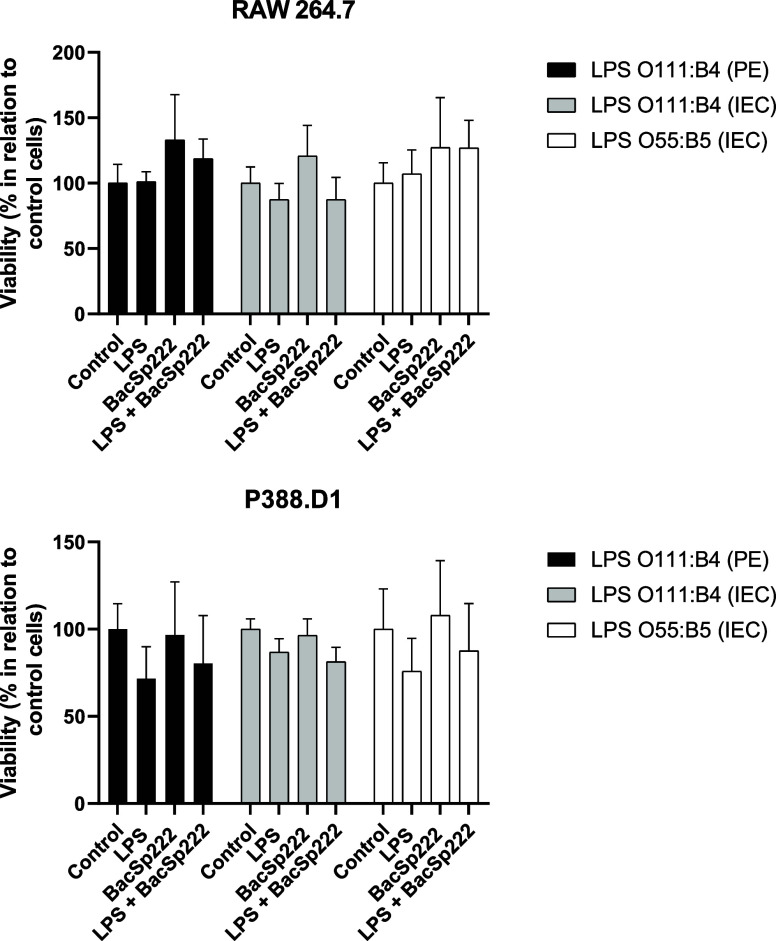
Metabolic activities of murine monocyte/macrophage cell lines simultaneously treated with LPS and BacSp222. The cells were cultured for 24 h in a medium (control) or a medium containing various types of LPS (100 ng/mL), BacSp222 (1 μM), or LPS with BacSp222. After stimulation, the metabolic activities of cells were measured using the MTT method. No significant differences were observed in the statistical analysis.

### BacSp222 Reduces the Ability of LPS to Stimulate the TLR4 Receptor and to Activate the Coagulation Pathway in the Assay

We performed further experiments to understand better the mechanism of inhibition of the pro-inflammatory response of cells to LPS in the presence of BacSp222. As is known, LPS is a compound recognized by one of the representatives of Toll-like receptors, specifically by the TLR4 receptor.^22^ We used genetically modified HEK-Blue cells that overexpress TLR4. After ligand binding to the receptor, the intracellular NF-κB-dependent signaling pathway is activated, leading to the expression of the reporter enzyme—secreted embryonic alkaline phosphatase (SEAP).

Our results showed that cells treated with LPS alone exhibited significantly higher levels of reporter protein expression than those treated simultaneously with both LPS and BacSp222 (Figure 4A). The most substantial difference in response was observed in cells treated with 0.2 ng/mL LPS, where the absorbance values were significantly reduced in the presence of 1 μM BacSp222 (absorbance was 0.461 and 0.094 AU for samples from only LPS-treated cells and LPS and BacSp222-treated cells, respectively), whereas the slightest difference was noted in cells treated with 5 ng/mL LPS and 1 μM BacSp222, with absorbance values indicating a slight decrease in receptor stimulation (0.564 vs 0.399 AU for samples from LPS-treated cells only and LPS and BacSp222-treated cells, respectively). These results suggest that the high ratio of bacteriocin molecules to LPS molecules leads to significant reduction in receptor activation.

**Figure 4:**
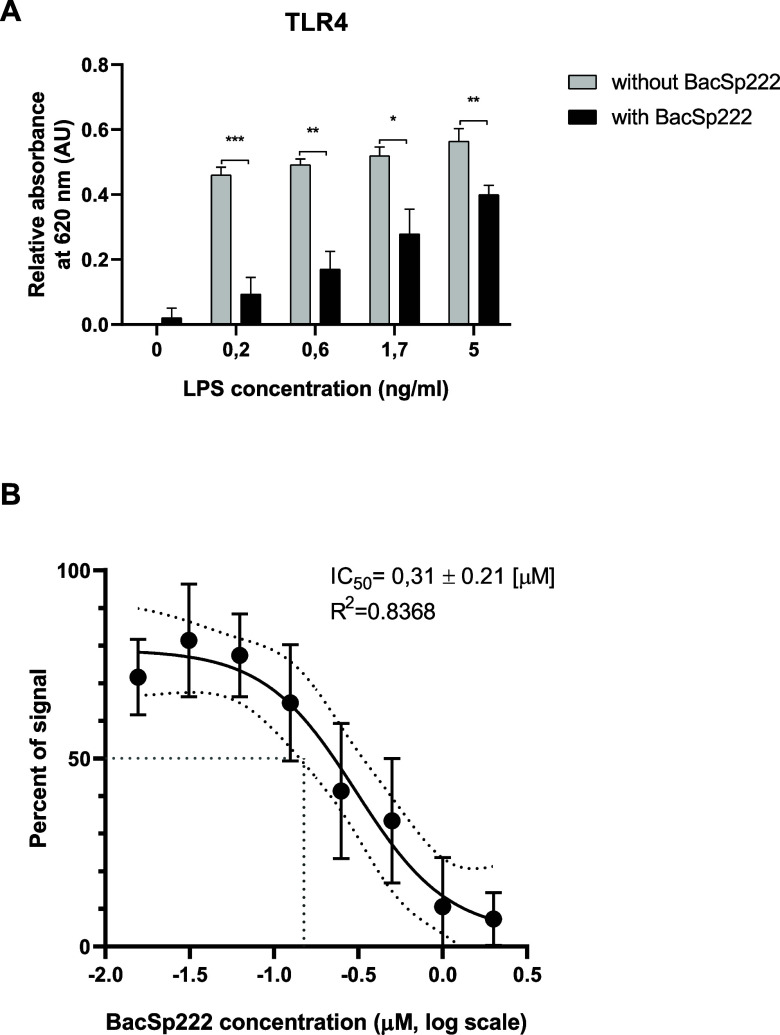
Analysis of the effect of BacSp222 on LPS-dependent activation of the TLR4 receptor. (A) HEK-Blue hTLR4 cells were incubated for 17 h in the medium or stimulated with different concentrations of LPS in the presence or absence of 1 μM BacSp222 or (B) in the media containing different concentrations of BacSp222 in the presence or absence of 0.2 ng/mL LPS. Then, the SEAP activity was measured in the postcultured media. (A) The bars represent the mean ± SD (*n* = 3), *p < 0.05, **p < 0.01, ***p < 0.001. (B) Data are shown as the percentage of absorbance for the LPS-treated samples. The dots represent the mean ± SD (*n* = 3). Four Parameter Logistic (4PL) Regression was fitted and IC_50_ was estimated.

To assess the inhibitory activity of the BacSp222 peptide on HEK-Blue TLR4 cells in a concentration-dependent manner, we independently stimulated the cells with a constant dose (0.2 ng/mL) of LPS and varying concentrations of BacSp222, ranging from 0.016 to 2 μM (Figure 4B). In the control experiment, we also tested these different concentrations of BacSp222 alone; however, such results were negative—none of the tested BacSp222 concentrations stimulated the TLR4 receptors. Moreover, we used sigmoidal curve fitting (4PL) to determine the half-maximal inhibitory concentration (IC_50_) of BacSp222, i.e., the concentration of BacSp222 required to inhibit TLR4 receptor activation by 50% in response to 0.2 ng/mL LPS. This BacSp222 concentration was 0.31 ± 0.21 μM (Figure 4B).

The separate and independent assay used to determine the reduction of the LPS biological potential after incubation with BacSp222 was a test. Briefly, this approach utilizes the ability of endotoxin to activate a proenzyme Factor C from amebocyte lysate and allows for spectrophotometric measurement of the released active enzyme using a chromogenic synthetic peptide substrate. Due to high specificity and sensitivity, this assay is widely used to detect trace amounts of LPS. As presented in Figure 5, preincubation of LPS with a large excess of BacSp222 reduced LPS activity by ca. 50%.

**Figure 5:**
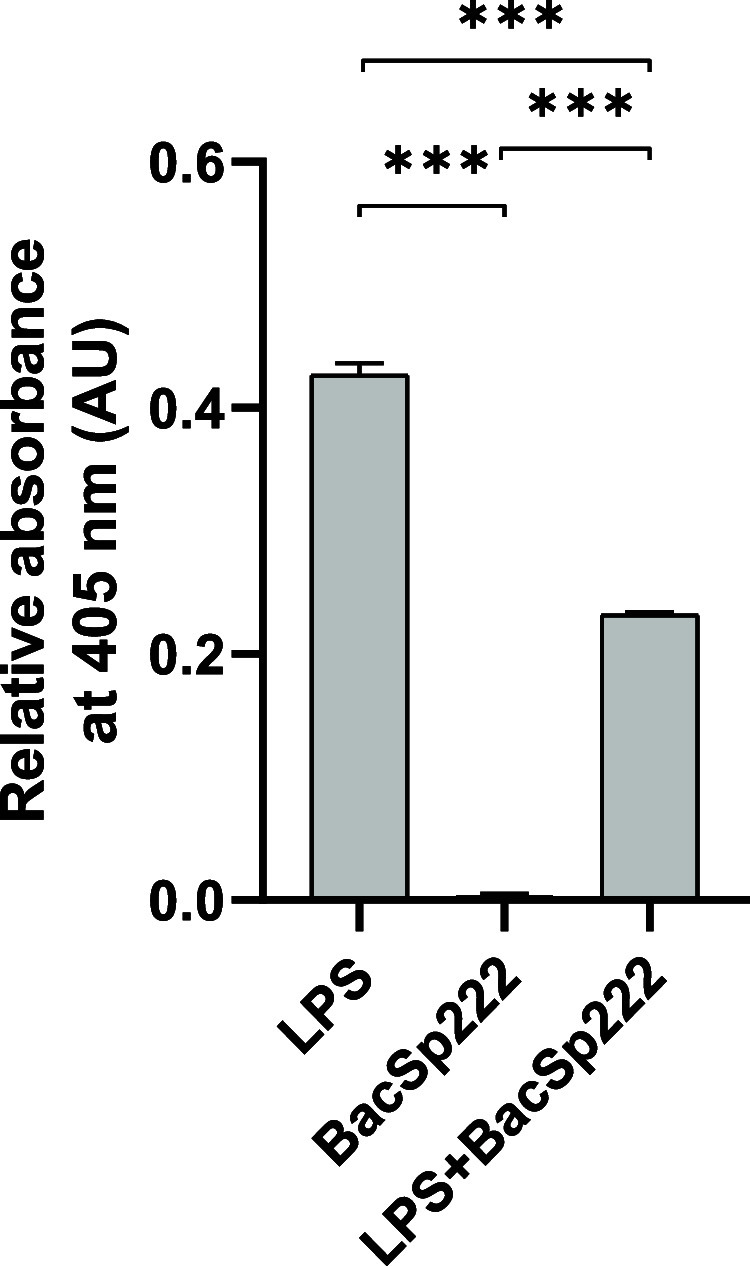
BacSp222 inhibits LPS biological activity. The bacteriocin was preincubated with LPS, and after this, the ability of LPS to activate the coagulation pathway was assayed in the assay. BacSp222 and LPS alone were used as controls. The details of the experiment are described in the Methods section; the bars represent the mean ± SD (*n* = 3), ***p < 0.001.

### LPS Does Not Decrease the Stimulation of TLR2/TLR6 Receptors by BacSp222

As demonstrated above, BacSp222 modulated the interaction between LPS and the TLR4 receptor. Our earlier studies confirmed that BacSp222 is a ligand for the TLR2/TLR6 heterodimer.^15^ Therefore, we investigated whether LPS impacts the interaction of BacSp222 with the TLR2/TLR6 heterodimer. For this purpose, we utilized genetically modified HEK-Blue cells that overexpress the TLR2/TLR6 heterodimer.

Our results indicated that even a substantial excess of LPS molecules compared to BacSp222 molecules (with LPS at a concentration of 5 μg/mL and BacSp222 at 0.05 μM) did not inhibit the TLR2/TLR6 stimulation by the bacteriocin. This was observed under both serum-enriched and serum-free conditions (Figure 6). In serum-free conditions, the presence of LPS did not significantly change the absorbance values (which were 0.281 and 0.372 AU for the samples collected from cells treated with BacSp222 alone and those treated with both LPS and BacSp222, respectively). At the same time, in the presence of serum, the absorbance values were 0.108 and 0.113 AU for the samples from cells treated with BacSp222 alone and those treated with both LPS and BacSp222, respectively. The results indicate that at the tested concentrations, LPS did not influence the interaction of BacSp222 with the TLR2/TLR6 heterodimer.

**Figure 6:**
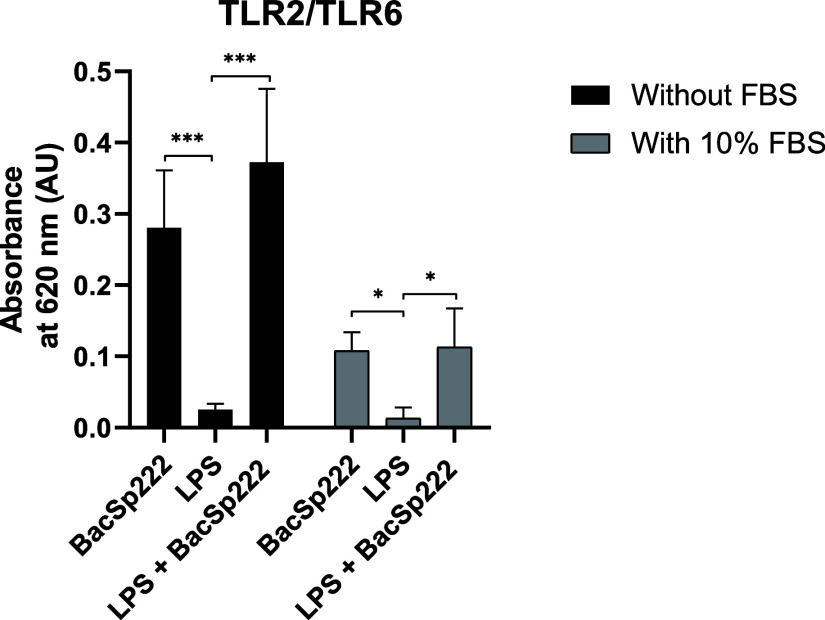
Analysis of the effect of LPS on BacSp222-dependent activation of the TLR2/TLR6 heterodimer. HEK-Blue hTLR2/TLR6 cells were incubated for 17 h in medium or stimulated with 0.05 μM BacSp222, 5 μg/mL LPS, or 0.05 μM BacSp222 + 5 μg/mL LPS in the presence or absence of FBS. Then, the SEAP activity was measured in postcultured media. The bars represent the mean ± SD (*n* = 3), *p < 0.05, ***p < 0.001 vs LPS-treated cells.

### BacSp222 Forms a Complex with LPS and Changes the Size of Its Aggregates

To verify the ability of BacSp222 and LPS to form a direct physical complex and to estimate its size, we applied gel filtration. The separations were performed in a volatile buffer that allowed further effortless electrophoretic analysis of fractions (after their evaporation), while the spectrophotometric signal was collected at 215 nm, capable of detecting both lipopolysaccharide and the peptide. Before separations, the column was calibrated using a set of proteins of known masses, as well as markers of total and void volumes. In applied conditions, BacSp222 elutes as a peak at 36 min (Figure 7A and B), and according to the calibration curve, this time corresponds to the molecular mass of 5.5 kDa, which is in agreement with an expected theoretical mass of the peptide.^7^ On the other hand, both examined LPS, from serotypes O111:B4 and O55:B5, eluted almost identically, and each preparation formed two separate peaks. The first peak, at 13 min, corresponds to ca. 700 kDa and is most probably a micellar (oligomeric) lipopolysaccharide form, while the second peak, eluting at 32 min, corresponding to 10.3 kDa, is, as we assume, a monomeric form (Figure 7A and B). This gel filtration image of LPS preparations is also consistent with theoretical data.^23^ Incubation of LPS with BacSp222 changed the observed chromatograms. The bacteriocin peak at 36 min disappears completely, while the peaks of LPS micellar forms shift from 13 to ca. 14 min, corresponding to the mass shift from 700 to ca. 620 kDa. The mentioned 14 min/620 kDa peak of LPS O55:B5 (IEC) incubated with bacteriocin was manually collected and analyzed electrophoretically under denaturing conditions, and the bacteriocin dissociated from the complex was visualized as a peptide band, unequivocally identified by N-terminal sequencing as BacSp222 (Figure 7C). On the other hand, it is worth emphasizing that incubation of both LPS serotypes with bacteriocin does not alter the monomeric LPS form; the 32 min/10.3 kDa LPS peak is the same and identical before and after incubation with BacSp222 (Figure 7A and B).

**Figure 7:**
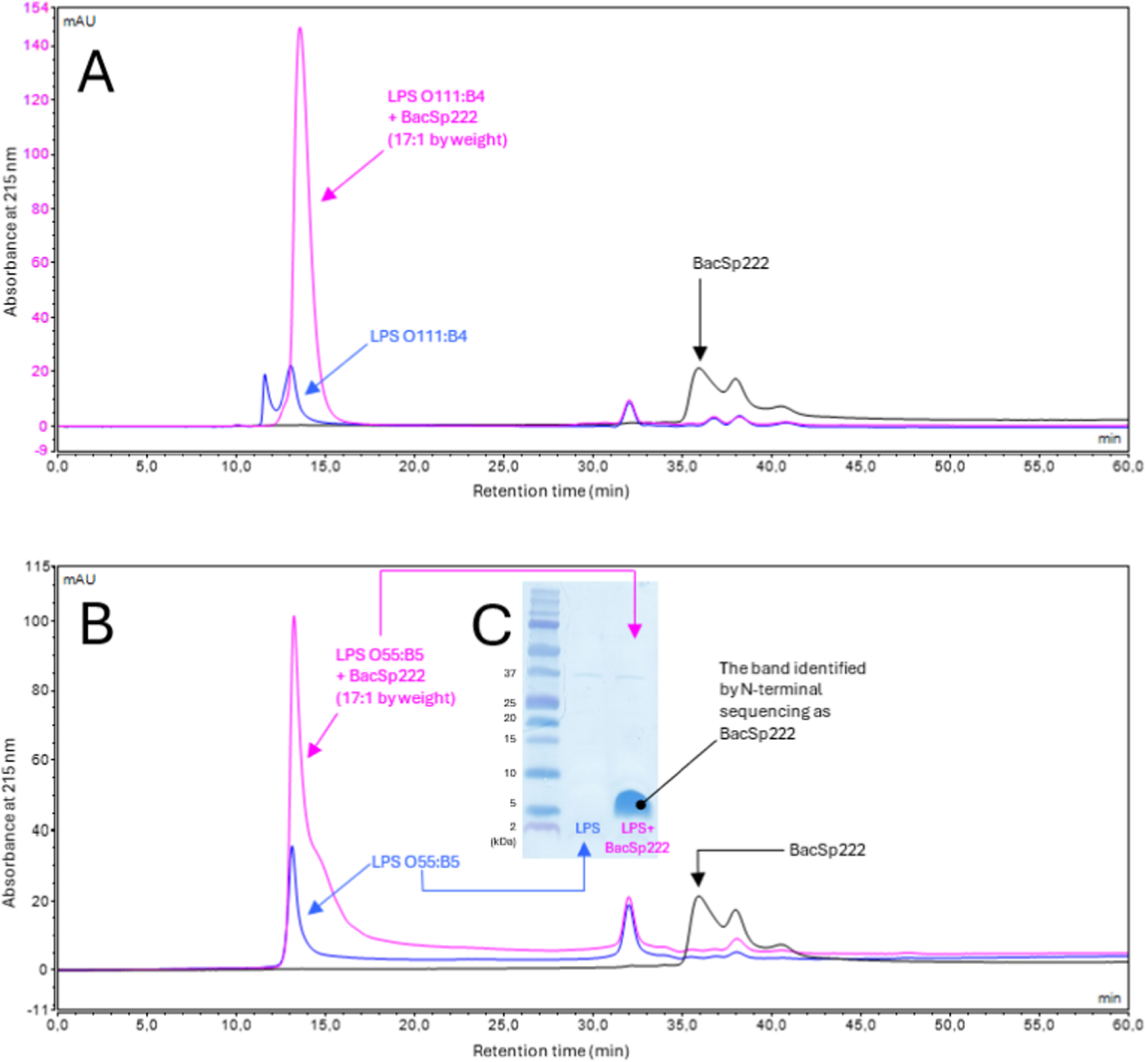
Gel filtration of different LPS (O111:B4 (PE) at panel A and O55:B5 (IEC) at panel B), BacSp222, and corresponding complexes between LPS and BacSp222. Insert C presents the SDS-PAGE separation of two indicated fractions containing LPS and LPS-BacSp222 complex. The peptide band denoted by a black dot and identified by N-terminal sequencing as BacSp222 unequivocally proves the formation of the complex. All chromatograms were recorded at 215 nm, while the remaining details of the experiments are described in the Methods section.

Independently, we confirmed the effect of bacteriocin on the size of micelles formed by LPS using the dynamic light scattering (DLS) method. Only LPS O55:B5 (IEC) appeared to be homogeneous, forming a micelle characterized by a hydrodynamic radius (HR) of 13.22 nm, and only this preparation was used for further evaluations (Supporting Information Figure S1). When LPS was mixed with BacSp222, the 13.22 nm micelle of LPS disappeared, and the obtained image depended on the mass proportions between the studied compounds (Supporting Information Figures S2A–C). At mass ratios of 10:1 and 1:1, the HR of LPS decreased to 8.70 and 7.56 nm, respectively. In contrast, mixing LPS with BacSp222 in a mass ratio 1:10 resulted in the formation of two entities with HR values of 1.42 and 7.56 nm. However, it should be kept in mind that this picture may be complicated by the fact that the BacSp222 molecule has a tendency to oligomerize in solutions, forming complex images dependent on the measured mass amount (Supporting Information Figure S2D).

To sum up, the gel filtration results that were obtained clearly prove that BacSp222 molecules form a direct complex with LPS, but only with their micellar forms. The precise stoichiometric proportions of such complexes were estimated in separate sections of this study concerning BacSp222 bactericidal potential inhibition.

The interaction between BacSp222 and LPS was also analyzed by using isothermal titration calorimetry (ITC). This method entails measurement of the heat resulting from the interaction between the reactants. The experimental system comprised a 93 μM BacSp222 solution, which was added in portions to the 48.5 μM LPS O55:B5 (IEC) solution. The experiments were conducted in PBS buffers differing in NaCl concentration: 137 and 500 mM. This different ionic strength allowed us to evaluate the significance of electrostatic interactions in the association of BacSp222 with LPS micelles. The greater the significance of electrostatic interactions in the observed interactions, the stronger they should be modulated by the change in the ionic strength. Reference measurements of BacSp222 solution, without LPS and irrespective of salt concentration, exhibited identical, near-zero enthalpies (open symbols in Figure 8). The presence of LPS in the measurement cell resulted in a significant heat response to BacSp222 injections (closed symbols in Figure 8). In the initial segment of the titration curve, a discernible endothermic or exothermic reaction is evident for low and high ionic strengths, respectively, which attain comparable enthalpy values for the molar ratio of BacSp222 to LPS of approximately 0.15. Subsequently, irrespective of the ionic strength, the reaction is endothermic and progresses to saturation, which is attained, contingent on the ionic strength, at BacSp222 to LPS molar ratios of 0.5 or 0.8 for low and high ionic strengths, respectively. This effect additionally indicates the direct interaction between BacSp222 and LPS molecules, while the nonmonotonic nature of the changes in the recorded thermal effects suggests the occurrence of several stages during their association: at least 3 steps for 137 mM NaCl and 2 steps for 500 mM NaCl. However, the complexity of the possible interaction mechanism precludes quantitative analysis of the obtained data. Nevertheless, the results obtained clearly indicate the dependence of the association of BacSp222 with LPS on the salt concentration, indicating that electrostatic interactions play a significant role in this process. The output raw data for the preparation of Figure 8 are provided in Supporting Information Figures S3 and S4.

**Figure 8:**
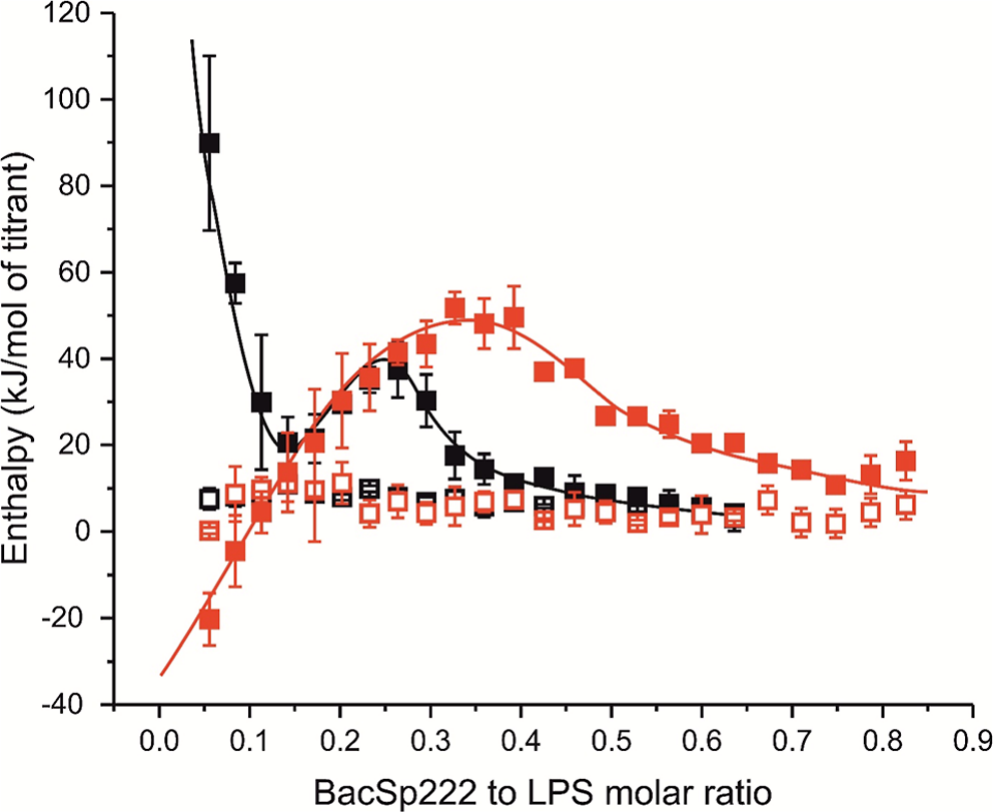
Isotherms of BacSp222 titrations against LPS. BacSp222 at 93 μM was added to 48.5 μM LPS O55:B5 (IEC) in PBS (black) or PBS containing 500 mM NaCl (red). Reference measurements (BacSp222 without LPS) are indicated by open symbols. The data are presented as the mean of two independent titrations ±SD. Continuous lines have been added only to make it easier to follow changes in thermal effects.

### LPS Inhibits Bactericidal Activity of BacSp222

The residual bactericidal activity of BacSp222 after incubation with LPS was measured by a radial diffusion assay toward four susceptible Gram-positive strains. Two 1:1 or 1:17 BacSp222:LPS mass ratios and three LPS preparations (O55:B5 (IEC), O111:B4­(IEC), and O111:B4 (PE)) were analyzed. The results presented in Figure 9 demonstrate that in the case of all four tested bacterial strains, the incubation of bacteriocin with equal weight of all LPS preparations only marginally reduces the bactericidal potential of the peptide. On the other hand, the usage of a 17-fold mass excess of LPS completely inhibited the killing potential of BacSp222. The only interesting exception was , where the excess of two LPS preparations (O55:B5 (IEC) and O111:B4 (PE) was able to only partially reduce the diameter of the inhibition zone (by ca. 50%). In the case of all bacteria and all mass amounts, no bactericidal activity was noticed in the case of each LPS preparation.

**Figure 9:**
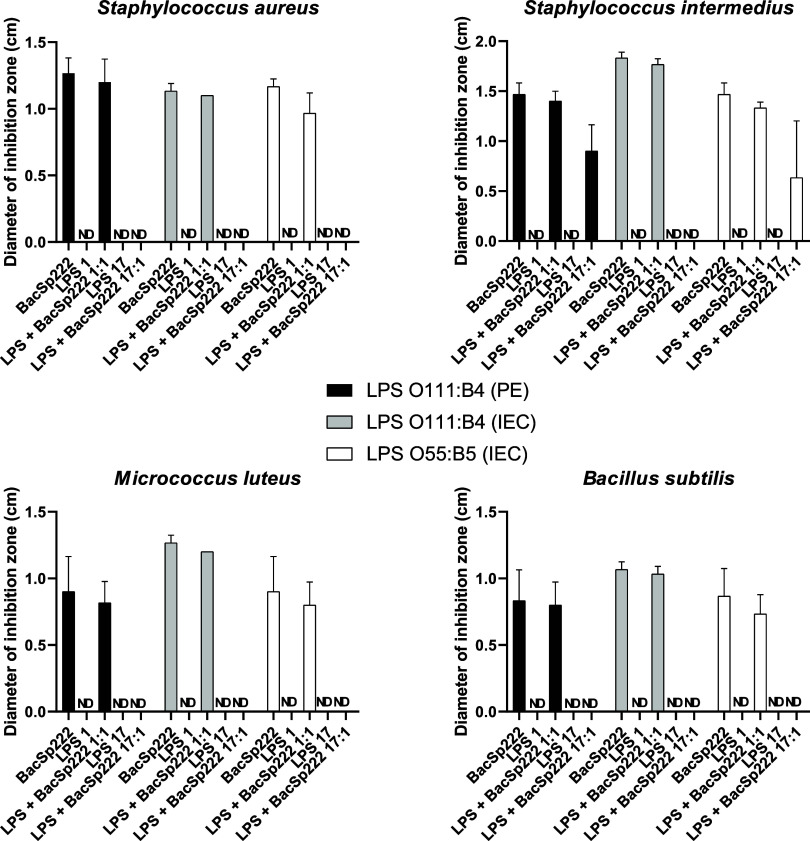
Analysis of the effect of LPS on the antibacterial activities of BacSp222. The antibacterial activity of the BacSp222, LPS, and BacSp222 + LPS solutions was checked against selected Gram-positive bacteria in a radial diffusion assay. The results were presented as the diameter of the bacterial growth inhibition zones. The bars represent the mean ± SD (*n* = 3).

The ability of an excess of LPS to completely inhibit BacSp222 bactericidal potential was utilized in a separate experiment to determine the stoichiometry of BacSp222 binding to LPS. In this experiment, an equal quantity of BacSp222 was incubated with different increasing amounts of two LPS preparations (O111:B4 (IEC) or O55:B5 (IEC)), and the residual bactericidal activity of bacteriocin was determined by radial diffusion and the linear relationship obtained allowed to arithmetically calculate the mass of particular LPS preparation necessary for complete inhibition of bacteriocin. The obtained stoichiometric ratios are presented in Figure 10 and demonstrate that ca. 10-fold molar excess of LPS O111:B4 (IEC) or 12-fold of LPS O55:B5 (IEC) (or ca. 17 and 21-fold for mass excess, respectively) is necessary to completely abolish the biological activity of BacSp222. Such results are in good agreement with the results from gel filtration presented above, where it was shown that BacSp222 binds only to oligomeric and micellar LPS forms.

**Figure 10:**
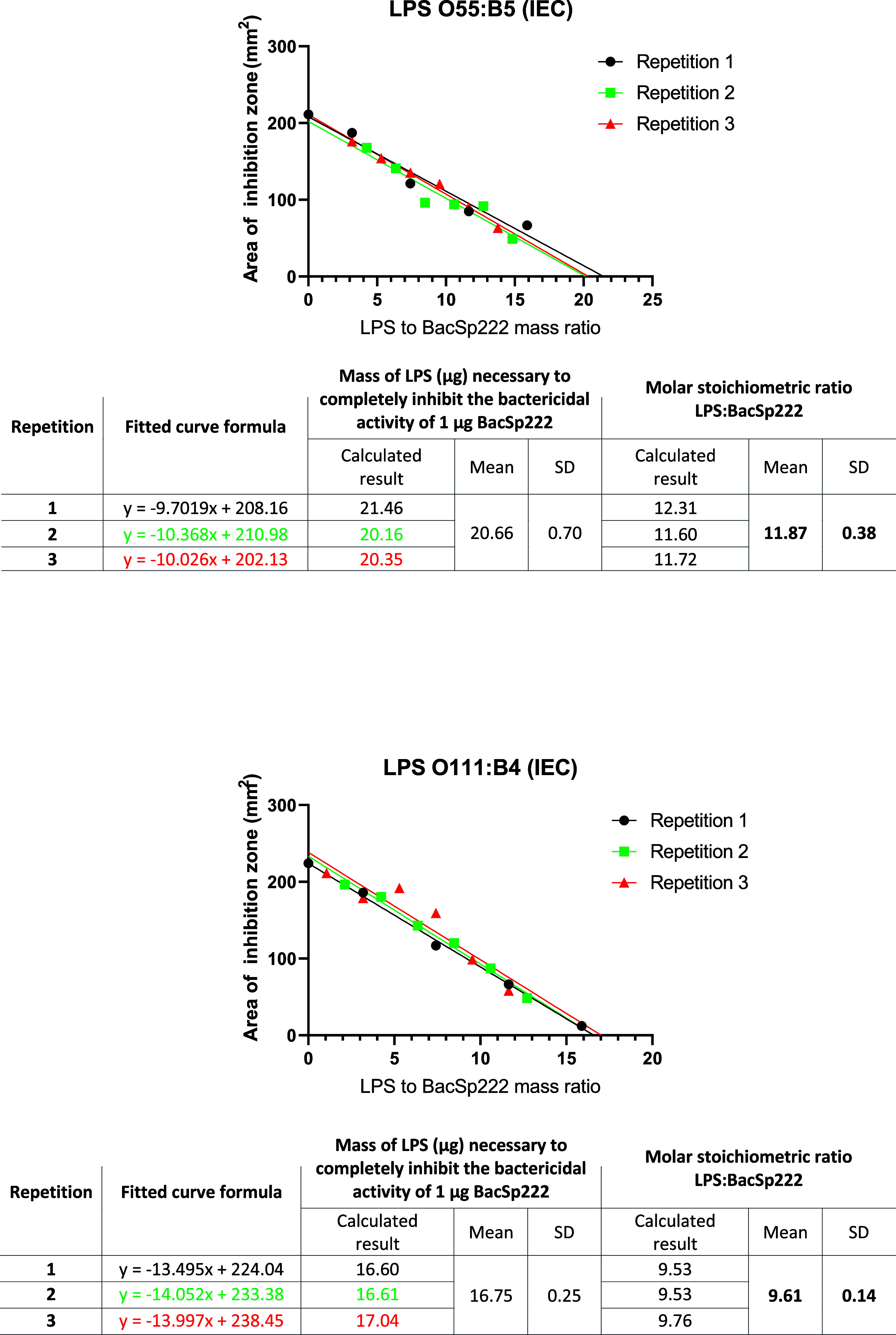
Evaluation of BacSp222 and LPS complex stoichiometry. An equal quantity of BacSp222 was preincubated with various amounts of LPS O111:B4 or O55:B5, and then the residual microbicidal activity of bacteriocin was evaluated using the radial diffusion assay. The areas of the emerged inhibition zones versus mass ratios were used to draw the appropriate relationships and to calculate the mean molar stoichiometric ratio of binding of particular LPS serotypes to the BacSp222 molecule. The details of the experiments are described in the Methods section.

### In the Insect In Vivo Model, the Activity of Hemolymph Phenoloxidase after Costimulation by BacSp22 and LPS Is Lower Than That after Separate Stimulation

The insect phenoloxidase activation model was applied to check the effect of BacSp222 on the LPS activity in vivo. LPS solution was injected into live larvae hemocel and activated hemolymph phenoloxidase system led to melanin formation, which was reflected by increased absorbance at 490 nm. This activation was inhibited when LPS was preincubated and coinjected with BacSp222. At a concentration of 3 μM, BacSp222 inhibited the activation of PO by LPS to about 50%. In comparison, the injection of bacteriocin alone resulted in PO activity at a similar level as the injection of PBS buffer (Figure 11, upper panel). Increasing the concentration of injected alone BacSp222 to 6 μM induced PO activity. Still, this increase was not additive to induction by LPS. In the presence of LPS and 6 μM BacSp222, the level of activation of PO was reduced in relation to induction caused by LPS alone (Figure 11, middle panel). Finally, injection of 30 μM BacSp222 alone resulted in significant activation of PO—to the same level as injection of LPS. However, the injection of LPS in the presence of BacSp222 was not additive to PO activation. On the contrary, after administration of LPS and 30 μM BacSp222, the PO activity was significantly reduced compared to the injection of both components separately. This result proves that in vivo LPS and BacSp222 hamper each other’s ability to induce PO in larvae.

**Figure 11:**
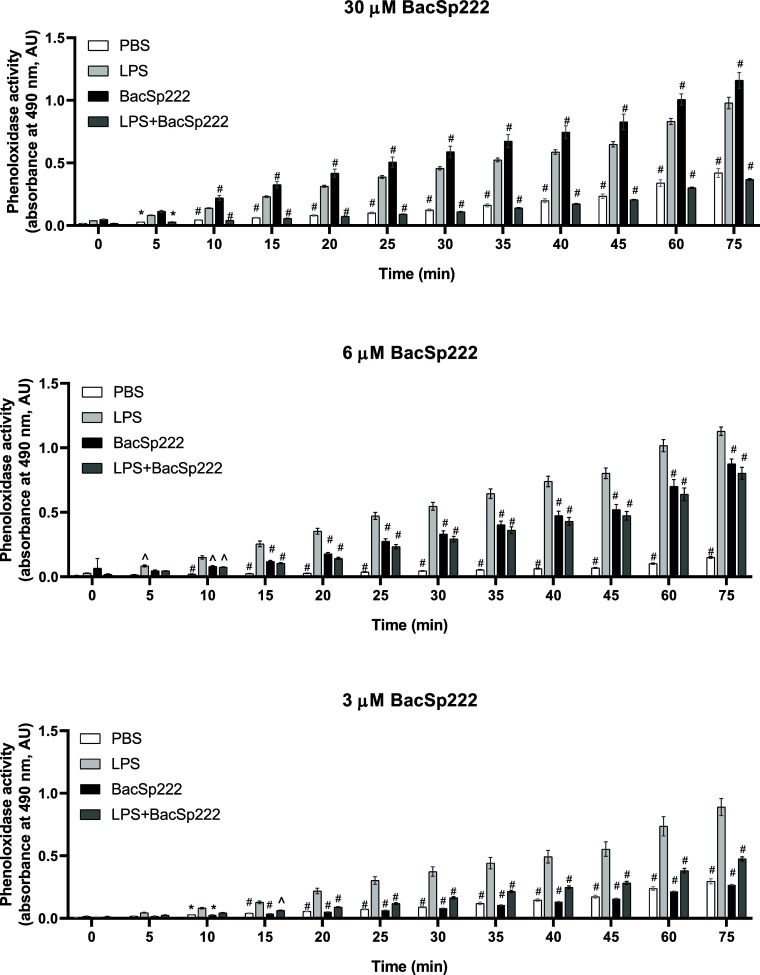
Activity of hemolymph phenoloxidase after stimulation of larvae by LPS in the presence of different amounts of BacSp222. The points represent the mean ± SD (*n* = 3), *p < 0.05, ^*p* < 0.01, #*p* < 0.001 vs LPS-treated larvae. The details of the experiments are described in the Methods section.

## Discussion

The present work concerns interactions between two distinctly different pro-inflammatory factors produced by bacteria. The first, LPS, is a crucial glycolipid constituent of the outer cell wall of Gram-negative bacteria and is mainly composed of three parts—lipid A, core, and O antigen.^24^ LPS plays a pivotal role as a permeability barrier that protects Gram-negative bacteria from the entry of small, hydrophobic molecules like antibiotics, bile salts, and detergents.^25^ Moreover, a vast and constant amount of LPS is released into the environment during bacteria death or division.^24^ Such free LPS can occur as a monomer or, after reaching the critical micellar concentration (CMC), in an aggregated micellar form. Both size, shape, and CMC of such aggregates depend on various factors such as pH, temperature, and the presence of divalent cations.^24,26,27^

The prevalence of LPS is particularly significant during bacterial infection as LPS has a strong immunogenic potential, being a key target of TLR4, an important innate immunity receptor. TLR4 responds strongly to the presence of lipid A, the most conserved part of LPS, leading to the activation of transcription factors NF-κB and IRF-3, and triggers the biosynthesis of various inflammation mediators such as IL-1β, TNF, IFNs, and IL-6.^24^ Additionally, LPS is an essential factor in the induction of ROS synthesis^28^ and NETs formation in neutrophils.^29^ Moreover, excessive systemic exposure to LPS can lead to severe sepsis, potentially resulting in the host’s death.^30^

On the other hand, BacSp222 belongs to a completely different class of bacterial biomolecules. It is a peptide bacteriocin produced by a Gram-positive zoonotic strain and possesses features of an antibacterial peptide as well as a virulence factor as it displays pro-inflammatory and cytotoxic activities toward eukaryotic cells.^7^ As we stated in the Introduction section, BacSp222 is a ligand of the TLR2/6 heterodimer and, through MyD88-dependent activation of NF-κB, stimulates the production of many pro-inflammatory cytokines.^15^ However, unlike LPS, BacSp222 cannot stimulate human neutrophils to ROS production and NETs formation.^16^ Moreover, unlike LPS, BacSp222 requires costimulation with IFN-γ to induce the production of selected cytokines and NO in macrophage-like cells.^16^

Our current work reveals that BacSp222 and LPS influence each other’s biological activities. Generally, LPS reduces the antimicrobial activity of BacSp222, while BacSp222 alters the pro-inflammatory properties of LPS, diminishing LPS’s ability to stimulate TLR4 and, consequently, to produce TNF and NO by macrophage-like cells. BacSp222 also inhibits LPS’s potential to mediate the clotting cascade in the assay. The TLR2/6 heterodimer effectively recognizes BacSp222 in the presence of LPS, in contrast to TLR4, which cannot recognize LPS in the presence of BacSp222. In an insect phenoloxidase activation model assay, the induction of PO, a key component of insect immune response, was significantly reduced after the injection of LPS and BacSp222 combined, compared to the independent injection of LPS or BacSp222, proving that in vivo LPS and BacSp222 mutually suppress each other’s pro-inflammatory activity. We also verified the interactions of BacSp222 and LPS by gel filtration and ITC. Although both molecules form a physical complex, it is essential to note that only the micellar form of LPS can interact with BacSp222.

Although the complex mechanism of binding of BacSp222 to LPS observed in calorimetric measurements precluded quantitative analysis, it is consistent with the data obtained for binding of other positively charged peptides to LPS.^31,32^ The postulated mechanism proposes that the initial step of the binding process involves interaction of the peptide with the negatively charged surface of LPS micelles. Subsequent processes are hypothesized to be related to the subsequent rearrangement of the complexes formed. In accordance with the aforementioned interpretation, the results in our study were obtained for high ionic strength for which the initial stage of binding was exothermic, subsequently transforming into endothermic. Conversely, the initial endothermic association process, observed at a low ionic strength, must be predominantly entropically driven. One potential explanation for this phenomenon is the release of water molecules from the hydration shells that occurs during the interactions.^33^

Proteins and peptides capable of recognizing and binding LPS as well as altering its biological activity are present in both higher organisms and bacteria. In higher organisms, these polypeptides are most often involved in the immune response. One of the best-known examples is LPS-binding protein (LBP).^34^ It is an acute-phase plasma protein of mammals that forms a complex with LPS, and this complex then binds to CD14 (cluster of differentiation 14). This event initiates a signaling pathway through TLR4/MD-2 and triggers the activation of immune response.^35,36^ In the absence of TLR4, MD-2 can also independently form a complex with LPS.^37^ Other examples of proteins of higher organisms that can interact with LPS include surfactant proteins (SP-A and SP-D) from lungs, hemoglobin, lysozyme, lactoferrin, heparin-binding proteins, and histones.^34^ Moreover, many cationic antimicrobial peptides can also bind to LPS and neutralize its pro-inflammatory properties.^34^ For instance, cathelicidins^34^ and beta-defensins^38^ found in vertebrates inhibit LPS-induced TLR4 activation. Similar activities were also observed in the case of frog magainin 2 and insect attacin.^34^

Regarding factors produced by bacteria, the canonical example of molecules interacting with LPS are polymyxins. They are cyclic cationic polypeptide antibiotics produced by and have a high affinity to LPS. Polymyxins are effective antibacterial drugs that interact with LPS in the outer and inner membranes of the cell wall, ultimately causing a lethal effect.^39^ Independently, through binding to lipid A, polymyxins effectively block the biological effects of LPS, including TLR4 activation, and this neutralizing effect is widely used to eliminate endotoxin contaminations in vitro and in vivo.^40–42^

Also, selected bacteriocins can interact with LPS. Colicins, a family of protein bacteriocins produced by , typically must cross the bacterial outer membrane to display bactericidal activity. Most colicins use porins and high-affinity receptors to cross the outer membrane and kill the target. However, for colicin N, there is no such high-affinity receptor, and this bacteriocin targets LPS instead. The receptor-binding domain of ColN binds to LPS near the membrane surface, and this interaction is required for its bactericidal activity.^43^

Nisin, a peptide lantibiotic produced by , is another example of a bacteriocin that could utilize LPS as a receptor. Nisin targets phosphate and pyrophosphate groups in LPS and forms a molecular complex in model membranes and bacterial outer membrane extracts. The interaction described above leads to membrane disruption. The level of membrane disruption is related to the origin of the bacteria and the type of LPS as its rough type is more susceptible to nisin.^19^

R-pyocins are bacteriophage tail-like bacteriocins produced by and display bactericidal activity by causing lysis after attachment to the cell surface of the target bacteria. However, these bacteriocins can also interact with LPS. There are several types of R-pyocins, each recognizing a different pattern in the LPS core and binding to it. Additionally, there is an association between R-pyocin susceptibility and the O-serotype of LPS.^44^ Other bacteriocins produced by , pyocin SD2 ^45^ and pyocin L1,^46^ which belong to the family of lectin-like bacteriocins, exhibit antimicrobial properties by recognizing the selected polysaccharide antigens of the target LPS molecule.

The examples described above demonstrate that the interaction between BacSp222 bacteriocin and LPS is no exception. However, the described studies on other bacteriocins focus not on their mutual interactions with LPS but instead on the bactericidal properties of bacteriocins. Our research revealed that BacSp222 and LPS form a physical complex and that this interaction influences the selected biological activities of both molecules. However, unlike other bacteriocins, the most distinctive feature of BacSp222 is its affinity only for LPS micelles. As we revealed in the previous study, the cationic charge of the BacSp222 molecule is essential to its antibacterial activity,^47^ while, on the other hand, in the presence of divalent cations, the charge of aggregates formed by LPS is negative.^26^ Additionally, just aggregates and not monomers of LPS play a crucial role in the activation of immune cells, and this phenomenon is related to forcing the right conformation of bisphosphorylated lipid A moieties.^48^ Thus, it can be assumed that the LPS molecule lacks one specific epitope responsible for binding to BacSp222, and observed interactions of both molecules, loss of antibacterial activity of BacSp222, and reduction in immunogenic potential of LPS are driven by less specific physicochemical interactions between the cationic polypeptide and negatively charged and membrane-mimicking micelles of self-assembling amphipathic aggregates of LPS. However, the details of these interactions require separate and more specialized studies.

## Conclusion

The results presented provide new data on the interactions between the bacteria themselves and, separately, the host during multispecies infections. BacSp222 and LPS are fundamentally different pro-inflammatory factors, produced by dissimilar bacteria, and capable of activating the host’s immune system through two distinct receptors. In theory, combined stimulation should increase the chances of effective activation of the immune response, resulting in a stronger pro-inflammatory reaction. However, the described interaction of BacSp222 with LPS and mutual suppression of their selected biological activities change this image and can result in a less effective elimination of bacteria from the infected niche.

## Methods

### Essential Macromolecules and Their Preparation

BacSp222 bacteriocin was isolated from 222 postculture medium and verified analytically, as described and illustrated in detail in previous papers.^15,16,47^ The purity (determined at over 99%) and peptide identity were checked by analytical reversed-phase high-pressure liquid chromatography (RP-HPLC), mass spectrometry, and N-terminal sequencing, while its concentration was determined by amino acid analysis. The possible contamination of the bacteriocin preparation by Gram-negative and/or Gram-positive endotoxins, lipopolysaccharide (LPS), and lipoteichoic acid (LTA), respectively, was carefully excluded by total phosphorus determination assays.

Three LPS preparations from serotypes O111:B4 and O55:B5 used in the study were purchased from Merck, Darmstadt, Germany. According to the manufacturer’s declaration, these preparations were purified by phenol extraction (PE, in case of O111:B4, cat. No. L2630) or by ion-exchange chromatography (IEC, in case O55:B5, cat. No. L4524 and O111:B4, cat. No. L3024). An ultrapure LPS from serotype O111:B4 was purchased from InvivoGen (San Diego, CA, USA, cat. No. tlrl-3pelps). For the assay, an O111:B4 Endotoxin Standard was used (Thermo, Waltham, MA, USA, cat. No. A39552S).

All solutions applied for experiments were prepared using endotoxin-free ultrapure laboratory water produced by the Purelab Maxima apparatus (ELGA LabWater, High Wycombe, UK).

### Cell Culture Conditions

The eukaryotic cells were cultured according to the methods described in previous publications.^15,10^ The murine monocyte/macrophage RAW 264.7 cell line (ATCC TIB-71) and the murine monocyte/macrophage P388.D1 cell line (ATCC CCL-46) were purchased from the American Type Culture Collection (ATCC, Manassas, VA, USA). Human TLR4/NF-κB/SEAP (HEK-Blue hTLR4) and human TLR2 + TLR6/NF-κB/SEAP (HEK-Blue hTLR2/TLR6) reporter HEK293 cells were obtained from InvivoGen (San Diego, CA, USA). The passages of all TLR reporter HEK293 cells ranged from 7 to 15. All cells used in experiments were cultured under the following conditions: 5% CO_2_, 37 °C, and >95% humidity. RAW 264.7 and P388.D1 cells were cultured in Dulbecco’s modified Eagle’s medium (DMEM) containing 4.5 g/L glucose (GIBCO, Paisley, UK) and 5% (v/v) fetal bovine serum (FBS, GIBCO, Paisley, UK), whereas HEK-Blue hTLR4 and HEK-Blue hTLR2/TLR6 were cultured in DMEM containing 4.5 g/L glucose, 10% (v/v) FBS, 50 units/mL penicillin, and 50 μg/mL streptomycin (both from GIBCO, Paisley, UK).

### Analysis of TNF Production by the Murine Monocyte–Macrophage Cells

RAW 264.7 and P388.D1 cells were seeded on 96-well plates at a density of 3 × 10^4^ cells per well in 100 μL of DMEM supplemented with 5% (v/v) FBS. After 24 h, the media were removed and replaced with (1) fresh DMEM (control) or with fresh DMEM containing (2) 100 ng/mL LPS O111:B4 (PE), or (3) 100 ng/mL LPS O111:B4 (IEC), or (4) 100 ng/mL LPS O55:B5 (IEC), or (5) 1 μM BacSp222, or (6) 100 ng/mL LPS O111:B4 (PE) + 1 μM BacSp222, or (7) 100 ng/mL LPS O111:B4 (IEC) + 1 μM BacSp222, or (8) 100 ng/mL LPS O55:B5 (IEC) + 1 μM BacSp222. After 6 h of stimulation, the concentrations of TNF in the postculture media were determined using the ELISA MAX Standard Set Mouse TNFα test (BioLegend, San Diego, CA, USA) according to the manufacturer’s instructions. Subsequently, the absorbance was measured at 450 nm using a Synergy H1 Hybrid plate reader controlled by Gene5 version 2.00.18 software (BIOTEK Instruments, Winooski, VT, USA). The results were shown as a percentage in relation to the cells treated by LPS alone, according to the formula

$$level\;of\;TNF\;\left(\%\;in\;relation\;to\;LPS-treated\;cells\right)=\frac{level\;of\;TNF\;in\;sample}{level\;of\;TNF\;in\;sample\;from\;LPS-treated\;cells}\times 100\%$$

### Analysis of NO Production by the Murine Monocyte–Macrophage Cells

RAW 264.7 and P388.D1 cells were seeded on 96-well plates at a density of 3 × 10^4^ cells per well in 100 μL of DMEM supplemented with 5% (v/v) FBS. After 24 h, the media were removed and replaced with (1) fresh DMEM containing 2% (v/v) FBS (control) or with fresh DMEM containing 2% (v/v) FBS and (2) 100 ng/mL LPS O111:B4 (PE), or (3) 100 ng/mL LPS O111:B4 (IEC), or (4) 100 ng/mL LPS O55:B5 (IEC), or (5) 1 μM BacSp222, or (6) 100 ng/mL LPS O111:B4 (PE) + 1 μM BacSp222, or (7) 100 ng/mL LPS O111/:B4 (IEC) + 1 μM BacSp222, or (8) 100 ng/mL LPS O55:B5 (IEC) + 1 μM BacSp222. After 24 h stimulation of cells, the postculture media were used to measure NO levels using the Griess assay. For this purpose, 50 μL of the postculture medium was mixed with 50 μL of a solution of 1% (w/v) sulfanilic acid and 0.1% (w/v) *N*-(1-naphthyl) ethylenediamine dihydrochloride (both from Sigma, St. Louis, MO, USA) in 2.5% (v/v) H_3_PO_4_. Then, the solutions were incubated for 10 min in the dark. The calibration curve was prepared using sodium nitrate (Sigma, St. Louis, MO, USA) in concentrations from 1.95 to 250 μM. The absorbance was measured at 545 nm using a Synergy H1 Hybrid plate reader controlled by Gene5 version 2.00.18 software (BIOTEK Instruments, Winooski, VT, USA). The results are shown as a percentage in relation to cells treated by LPS alone, according to the formula

$$level\;of\;NO\;\left(\%\;in\;relation\;to\;LPS-treated\;cells\right)=\frac{level\;of\;NO\;in\;sample}{level\;of\;NO\;in\;sample\;from\;LPS-treated\;cells}\times 100\%$$

### Analysis of the Mitochondrial Activity (MTT Assay)

RAW 264.7 and P388.D1 cells were cultured and stimulated as described above in the section concerning the determination of NO production. The MTT assay was applied to measure the metabolic activity of the cells, indicating their viability. This assay was performed according to the standard protocol, and the absorbance was measured at 545 nm using a Synergy H1 Hybrid plate reader controlled by Gene5 version 2.00.18 software (BIOTEK Instruments, Winooski, VT, USA). The cell viability was calculated according to the formula

$$viability\;\left(\%\;in\;relation\;to\;control\;cells\right)=\frac{absorbance\;of\;sample\;from\;treated\;cells}{absorbance\;of\;sample\;from\;control\;cells}\times 100\%$$

### Stimulation of HEK-Blue Cells with hTLR4 and hTLR2/TLR6 Receptors

HEK-Blue hTLR4 cells were seeded on a 96-well plate at density 2.5 × 10^4^ cells per well in (1) 100 μL DMEM containing 10% (v/v) FBS, 50 units/mL penicillin, 50 μg/mL streptomycin (control) or in 100 μL DMEM containing 10% (v/v) FBS, 50 units/mL penicillin, 50 μg/mL streptomycin with (2) LPS O111/B4 (ultrapure) at concentration 0.2, 0.6, 1.7, and 5 ng/mL, or (3) 1 μM BacSp222, or (4) 1 μM BacSp222 in the presence of LPS O111:B4 (ultrapure) at concentration 0.2, 0.6, 1.7, and 5 ng/mL. After 17 h, the media were collected to detect secreted embryonic alkaline phosphatase (SEAP) according to the protocol described below.

To determine the inhibitory activity of the bacteriocin on HEK-Blue hTLR4 cells in a concentration-dependent manner, the cells were seeded on a 96-well plate at density 2.5 × 10^4^ cells per well in 100 μL DMEM containing 10% (v/v) FBS, with (1) BacSp222 at concentration 2 μM, 1 μM, 0.5 μM, 0.25 μM, 0.125 μM, 0.063 μM, 0.031 μM, 0.016 μM, and 0 μM (control), or (2) LPS O111:B4 (ultrapure) at concentration 0.2 ng/mL in the presence of BacSp222 in concentrations identical as in the previous point. After 17 h, the media were collected to detect SEAP according to the protocol described below.

HEK-Blue hTLR2/TLR6 cells were seeded on a 96-well plate at density 2.5 × 10^4^ cells per well in (1) 100 μL of DMEM containing 50 units/mL penicillin, 50 μg/mL streptomycin (control) or in 100 μL of DMEM containing 50 units/mL penicillin, 50 μg/mL streptomycin and (2) 0.05 μM BacSp222, (3) 5 μg/mL LPS (ultrapure, O111/B4), or (4) 0.05 μM BacSp222 + 5 μg/mL LPS (ultrapure, O111/B4). Each stimulation was performed in the presence or absence of 10% (v/v) FBS. After 17 h, the media were collected to detect secreted embryonic alkaline phosphatase (SEAP) according to the protocol described below.

### Secreted Embryonic Alkaline Phosphatase Detection

Measurement of the activity of SEAP, secreted by the HEK-Blue cells to the media, was conducted as described previously.^15^ Briefly, 10 μL of postculture medium was added to 90 μL Cell Culture Medium for SEAP Detection (InvivoGen, San Diego, CA, USA). Then, the plate was incubated at 37 °C for 1 h in the dark, and the absorbance was measured at 620 nm using a Synergy H1 Hybrid microplate reader controlled by Gene5 version 2.00.18 software (BIOTEK Instruments, Winooski, VT, USA). The relative absorbance was calculated according to the formula

relativeabsorbance=absorbancefromsample−absorbancefromthecontrolsample

whereas the percentage of absorbance for the LPS-treated samples determined for HEK-Blue cells treated with 0.2 ng/mL LPS and various concentrations of BacSp222 was calculated according to the formula

$$percent\;of\;signal=\frac{relative\;absorbance\;of\;sample\;from\;treated\;cells}{elative\;absorbance\;of\;sample\;from\;LPS-treated\;cells}\times 100\%$$

### Gel Filtration and Dynamic Light Scattering Experiments

Gel filtration was conducted on a Superdex 200 Increase 10/300 GL (Cytiva, Marlborough, MA, USA) column using an UltiMate 3000 HPLC apparatus (Thermo, Waltham, MA, USA). The separations were performed at room temperature in 50 mM NH_4_COOH pH 5.8 at a flow rate of 0.6 mL/min. The spectrophotometric detection was conducted at 215 and 280 nm, and before separations, the column was calibrated using tryptophan (204 Da), concanavalin (75 kDa), aldolase (158 kDa), ferritin (440 kDa), and Blue Dextran 2000 (2 MDa). The following samples were separated: (1) 375 μg of LPS O55:B5 (IEC), (2) 375 μg of LPS O111:B4 (PE), (3) 75 μg of BacSp222, (4) 375 μg of LPS O55:B5 (IEC) + 75 μg BacSp222, and (5) 375 μg of LPS O111:B4 (PE) + 75 μg BacSp222. All above solutions were prepared in phosphate-buffered saline (PBS) without Ca^2+^ and Mg^2+^ (Gibco, Paisley, UK) and were preincubated before separation for 15 min at 37 °C. The fractions containing LPS O55:B5 alone and LPS-BacSp222 complex, visible as peaks eluting at 12.5–14 min and 12.5–16 min, both marked by arrows in Figure 7, were manually collected, freeze-dried, and subjected to sodium dodecyl sulfate polyacrylamide gel electrophoresis (SDS-PAGE) under reducing conditions.^49^ After separation, the gel was electrotransferred on a 0.22 μm pore-size polyvinylidene difluoride (PVDF) membrane (Immobilon-PSQ, Merck, Darmstadt, Germany), stained with Coomassie blue and the band of BacSp222 peptide was cut out from the membrane and subjected to deblocking—chemical removal of N-terminal formyl-methionine by cyanogen bromide—as described in a previous paper.^16^ After deblocking, the PVDF-bound peptide was identified by N-terminal amino acid sequence determination using a PPSQ-31 A automatic protein sequencer (Shimadzu, Japan).

For dynamic light scattering experiments, (1) the solution of PBS without Ca^2+^ and Mg^2+^ or PBS without Ca^2+^ and Mg^2+^ with (2) 50 μg/mL LPS O55:B5 (IEC), (3) 50 μg/mL LPS O111:B4 (IEC), (4) BacSp222 at concentration 5, 50, and 500 μg/mL, or (5) 50 μg/mL LPS O55/B5 (IEC) + BacSp222 at concentration 5, 50, and 500 μg/mL were incubated for 15 min at 37 °C. Then, the hydrodynamic radius of the LPS and BacSp222 molecules, as well as LPS-BacSp222 complexes, was determined by dynamic light scattering (DLS) on the Prometheus Panta apparatus (NanoTemper, Munich, Germany). Prometheus Standard Capillaries (NanoTemper, Munich, Germany) were used in the experiments, and the measurements were taken at 37 °C. The results were processed by the instrument software (Prometheus Panta Software).

### Determination of the Residual Biological Activity of LPS by a Assay

The residual biological activity of LPS after preincubation with BacSp222 was evaluated by using a commercial assay kit. The reaction was performed for 4 μg of BacSp222, which was preincubated with 3 pg of LPS O111:B4 standard for 15 min at 37 °C in a final volume of 60 μL of PBS without Ca^2+^ and Mg^2+^. Simultaneously and independently, the same quantity of BacSp222 and LPS as above was preincubated separately as controls. After preincubation, the residual biological activity of endotoxin in all samples was determined using a spectrophotometric Pierce Chromogenic Endotoxin Quant Kit (Thermo, Waltham, MA, USA). The absorbance was measured at 405 nm using a microplate reader Synergy H1 Hybrid plate reader controlled by Gene5 version 2.00.18 software (BIOTEK Instruments, Winooski, VT, USA).

### Determination of the Residual Bactericidal Activity of Bacteriocin by Radial Diffusion Assay

The residual bactericidal activity of BacSp222 after preincubation with different LPS serotypes and at different mass proportions was determined using radial diffusion assay toward the following bacteria: ATCC 25923, ATCC 29663, ATCC 6633, and ATCC 4698. BacSp222 at final concentrations of 10 μM (for and ) or 50 μM (for and ) was preincubated in PBS without Ca^2+^ and Mg^2+^ for 15 min at 37 °C with LPS O55:B5 (IEC) or O111:B4 (PE and IEC) at proportions equivalent to 1:1 or 1:17 mass ratios (BacSp222:LPS). After preincubation, 5 μL aliquots of the solutions were dispensed as drops at different sites of soft Mueller–Hinton Broth II (MHBII, Merck, Darmstadt, Germany) plates containing 0.75% (w/v) agarose and a suspension of the tested bacteria strains. After liquid preparations were entered into the solid medium, the plates were incubated at 37 °C for 16 h, and the antibacterial activity of the studied samples was determined as the diameter of the bacterial growth inhibition zone. In each experiment, the appropriate controls were applied on the plate, and they comprised adequate amounts of BacSp222 and LPS not incubated together.

### Evaluation of BacSp222-LPS Complex Stoichiometry

An equal quantity of 2.4 μg of BacSp222 was preincubated for 20 min at 37 °C with various amounts of LPS O111:B4 (IEC) or O55:B5 (IEC) ranging from 7.5 to 47.4 μg in the final volume of 11.3 μL of PBS without Ca^2+^ and Mg^2+^. After this, 5 μL aliquots of all solutions were used to determine the residual microbicidal activity of bacteriocin using the radial diffusion assay described in the previous section. The results were expressed as areas of the inhibition zones and used to outline the relationship of the inhibition zone area to the mass ratio of LPS to BacSp222. The point where the line intersects the *X*-axis indicates the mass of LPS necessary for complete inhibition of bacteriocin activity (Figure 10). Obtained values allowed to calculate the mean molar stoichiometric ratio of binding of particular LPS serotypes with BacSp222, assuming the molecular masses of BacSp222 molecule at 5.922 kDa^7^ and LPS monomer at 10.300 kDa (estimated in this work by gel filtration and consistent with other sources^23^).

### Calorimetry of the Interaction of BacSp222 and LPS

The interaction of the bacteriocin with LPS was measured using an Affinity ITC microcalorimeter (TA Instruments, New Castle, DE, USA). Measurements were performed in duplicate at 37 °C in PBS or PBS containing 500 mM NaCl. Before the experiments, all solutions were degassed under a vacuum for 10 min. Titration was performed by adding 20–25 injections of a 93 μM solution of BacSp222 in a volume of 3 μL to a measuring cell containing a 48.5 μM solution of LPS O55:B5 (IEC). In the reference measurements, the cells contained only the appropriate buffer. The interval between injections was 3.5 min, and the stirring speed of 125 rpm was maintained throughout the experiment. Data were analyzed using the apparatus manufacturer’s software. The molar concentrations were calculated assuming molecular weights of 5.922 and 10.3 kDa, for BacSp222 and LPS monomer, respectively. The data are presented as the mean of two independent titrations ±SD.

### Insects and In Vivo Experiments

The experiments were based on a previously published protocol.^50^ The greater wax moth () was reared in the dark on honeybee nest debris at 28 °C and 70% humidity. Last instar larvae were used for the experiments. To test the activation of the phenoloxidase (PO) system by LPS, larvae were divided into groups of 10 insects. One group was injected with PBS buffer without Ca^2+^ and Mg^2+^, the other one with 1 μg of LPS O55:B5 (IEC) diluted in PBS, and the next one with the mixture of 1 μg of LPS and BacSp222 at concentrations 3, 6, or 30 μM. The last group was treated with the bacteriocin alone at the same concentrations. Before injection, all prepared solutions were preincubated for 30 min at 30 °C for possible complex formation, while the puncture surface of each larva was disinfected with 70% ethanol. Injection was done in the last but one proleg by a Hamilton syringe at a volume of 5 μL. After injection, the larvae were placed in Petri dishes on sterile filter paper. Bleeding larvae were excluded from the experiment. The larvae were cooled for hemolymph collection, their body surface was sterilized with 70% ethanol, and the larvae were punctured with a sterile scalpel. The hemolymph was collected into Eppendorf tubes containing a few phenylthiourea crystals to prevent melanization induced by larvae injury. The hemolymph was then centrifuged at 200*g* for 5 min at 4 °C to pellet hemocytes and then at 20,000 g for 10 min at 4 °C to eliminate any debris. The collected hemolymph was stored at −20 °C until use.

### Determination of Hemolymph Phenoloxidase Activity

The assay was performed similarly to the previous papers.^51,52^ The hemolymph was diluted 5 times in apyrogenic water, and 2 μL of this dilution was added to 18 μL of buffer A (50 mM Tris–HCl pH 7.4, 150 mM NaCl, 5 mM CaCl_2_) in a 96-well plate. After 20 min of preincubation at room temperature, 180 μL of a 2 mM solution of 3,4-dihydroxy- *l* -phenylalanine (l-DOPA) in buffer B (50 mM Na_2_HPO_4_, 50 mM NaH_2_PO_4_; pH 6.5) was added, and then, the absorbance at 490 nm was measured immediately (time 0) at the indicated time-points. Before measurements, the plate was kept at 30 °C. The blank sample contained water instead of hemolymph.

### Data Presentation and Statistical Analysis

All experiments were conducted three times independently, except for the DLS and ITC measurements, which were performed once and twice, respectively. The results were presented as mean ± standard deviation (SD). For experiments concerning measurements of TNF, NO, cellular metabolic activity, TLRs activation, and LAL assay, the statistical significance of differences between the particular result and control was calculated using a two-way ANOVA with Tukey’s multiple comparison test as a posthoc test and shown in figures as asterisks *, **, or ***, indicating *p* < 0.05, *p* < 0.01, and *p* < 0.001, respectively. In the case of phenoloxidase activity measurements, an ordinary two-way ANOVA with Dunnett’s multiple comparison test was applied and shown in figures as asterisks: *,^ or #, for *p* < 0.05, *p* < 0.01, or *p* < 0.001, respectively. The sigmoidal, four-parameter logistic (4PL) curve fitting was used to assess the dependence of the TLR4 receptor stimulation signal by LPS in the presence of different concentrations of BacSp222.

## Supplementary Material



## Data Availability

Source data are available at the Open Research Data Repository for Kraków Universities (RODBUK), accessible at https://uj.rodbuk.pl/dataverse/wbbib.

## Notes

Analysis of the hydrodynamic radius of various types of LPS using the DLS method, effect of coincubation of various mass proportions of LPS and BacSp222 on the hydrodynamic radius of the analyzed molecules evaluated by the DLS method, ITC data of BacSp222 titration to buffers, and ITC data of BacSp222 interactions with LPS (PDF)
