# Supplementary material for: Interactions between Lipopolysaccharide and Peptide Bacteriocin BacSp222 Influence Their Biological Activities
Source: ACS Infect Dis. 2025 Jul 8;11(8):2116–30. doi: 10.1021/acsinfecdis.5c00066 (PMC12340957; doi:10.1021/acsinfecdis.5c00066)
Supplement: Supplementary file 1 [file id5c00066_si_001.pdf]

SUPPORTING INFORMATION

Interactions between lipopolysaccharide and peptide bacteriocin BacSp222 influence their biological activities

Justyna Śmiałek-Bartyzel [1,2], Monika Bzowska [3], Alicja Frączek [1,2], Iwona Wojda [4], Renata Mężyk-Kopeć [3], Piotr Bonarek[5], Artur Blat [1,6], Jan Rak[2], Paweł Mak [2]*

[1] Doctoral School of Exact and Natural Sciences, Jagiellonian University, Łojasiewicza 11 St., 30-348 Kraków, Poland
[2] Department of Analytical Biochemistry, Faculty of Biochemistry, Biophysics and Biotechnology, Jagiellonian University, Gronostajowa 7 St., 30-387 Kraków, Poland
[3] Department of Cell Biochemistry, Faculty of Biochemistry, Biophysics and Biotechnology, Jagiellonian University, Gronostajowa 7 St., 30-387 Kraków, Poland
[4] Department of Immunobiology, Institute of Biological Sciences, Maria Curie-Sklodowska University, Akademicka 19, 20-033 Lublin, Poland.
[5] Department of Physical Biochemistry, Faculty of Biochemistry, Biophysics and Biotechnology, Jagiellonian University, Gronostajowa 7 St., 30-387 Kraków, Poland
[6] Małopolska Centre of Biotechnology, Jagiellonian University, Gronostajowa 7A St., 30-387 Kraków, Poland

* Corresponding author, e-mail address: pawel.mak@uj.edu.pl

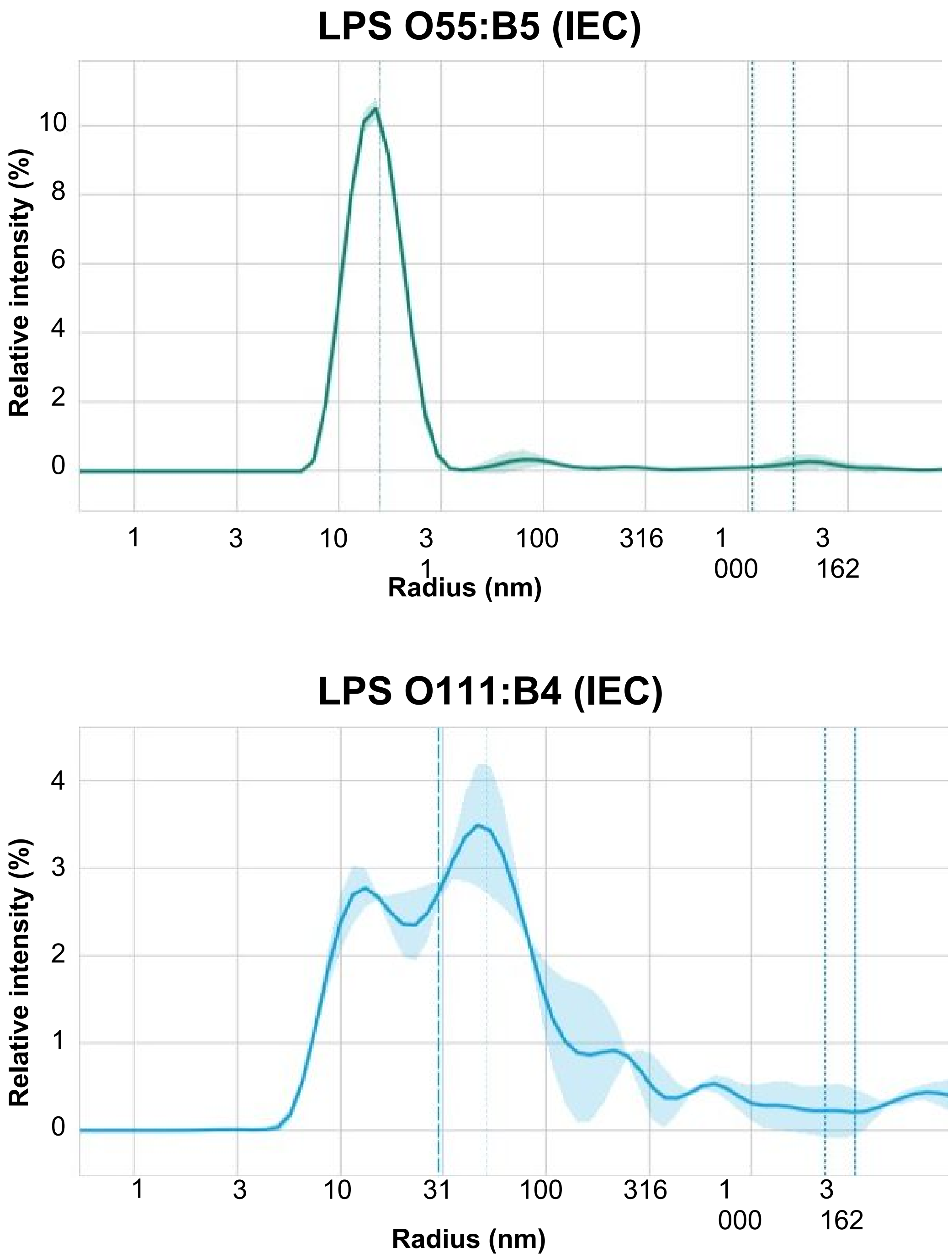

Figure S1. Analysis of the hydrodynamic radius of various types of LPS using the DLS method.

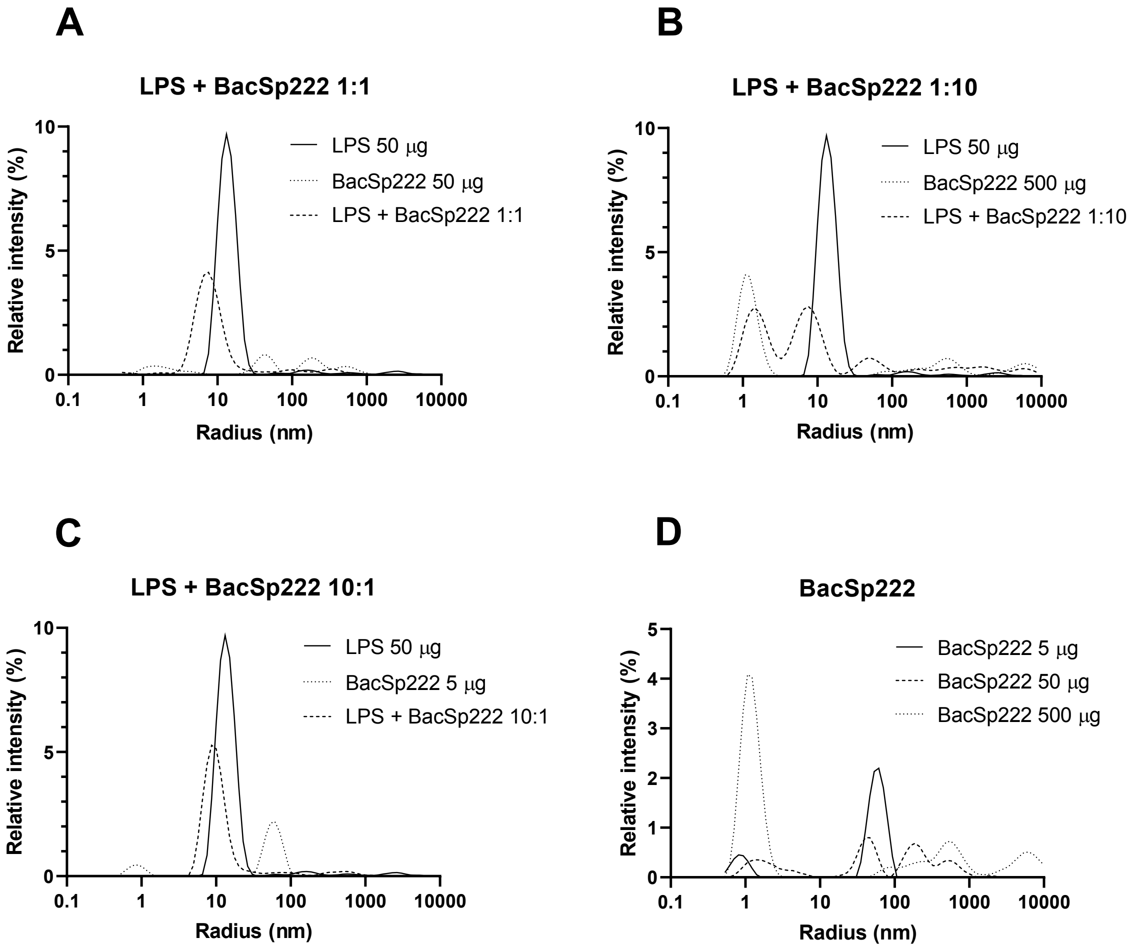

Figure S2. The effect of coincubation of various mass proportions of LPS O55:B5 (IEC) and BacSp222 on the hydrodynamic radius of the analyzed molecules evaluated by the DLS method.

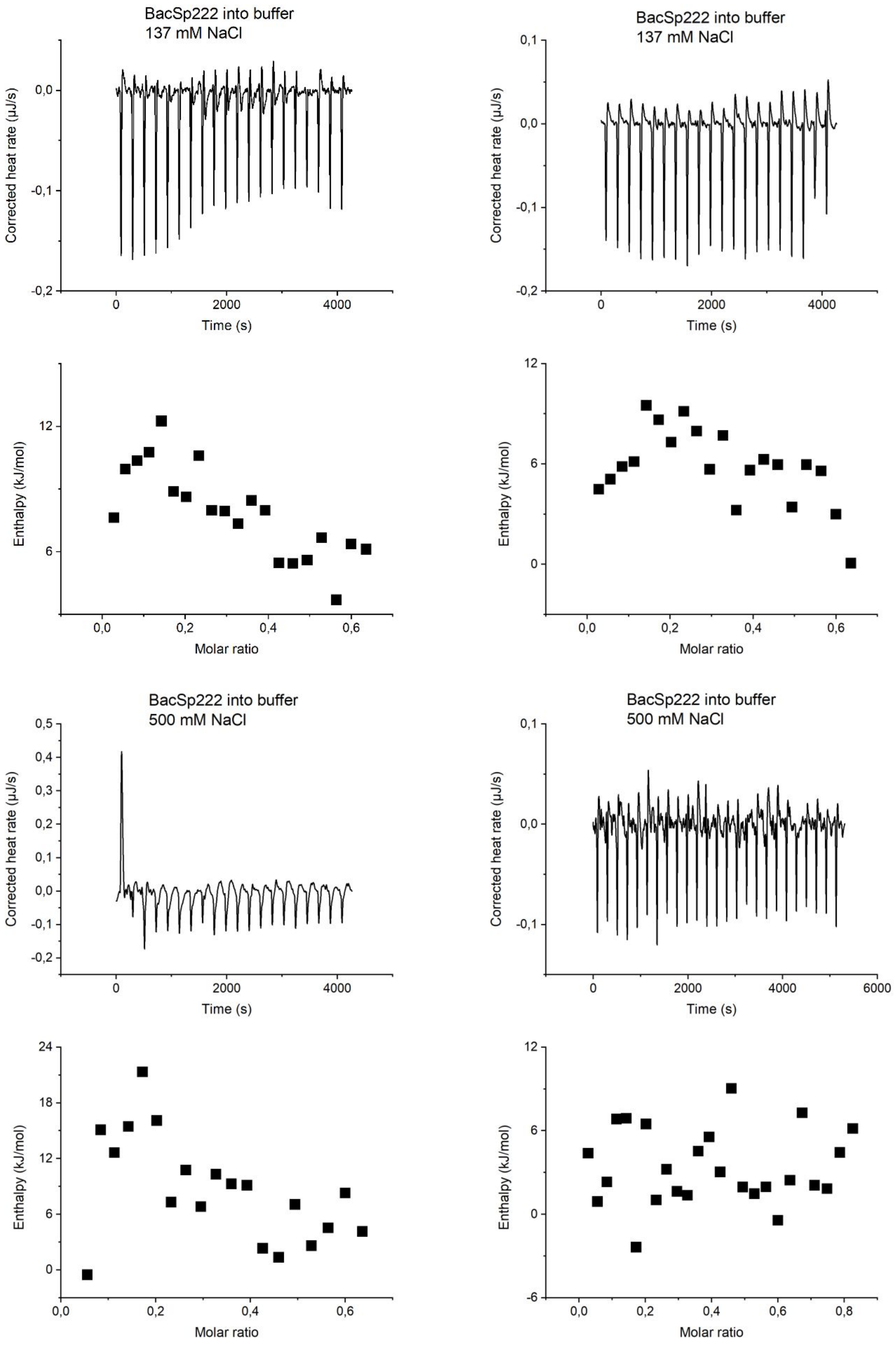

Figure S3. ITC data of BacSp222 titration to buffers. Titration was performed at 37 °C by adding 20-25 injections of 93 µM solution of BacSp222 in a volume of 3 µl to a measuring cell containing the buffer.

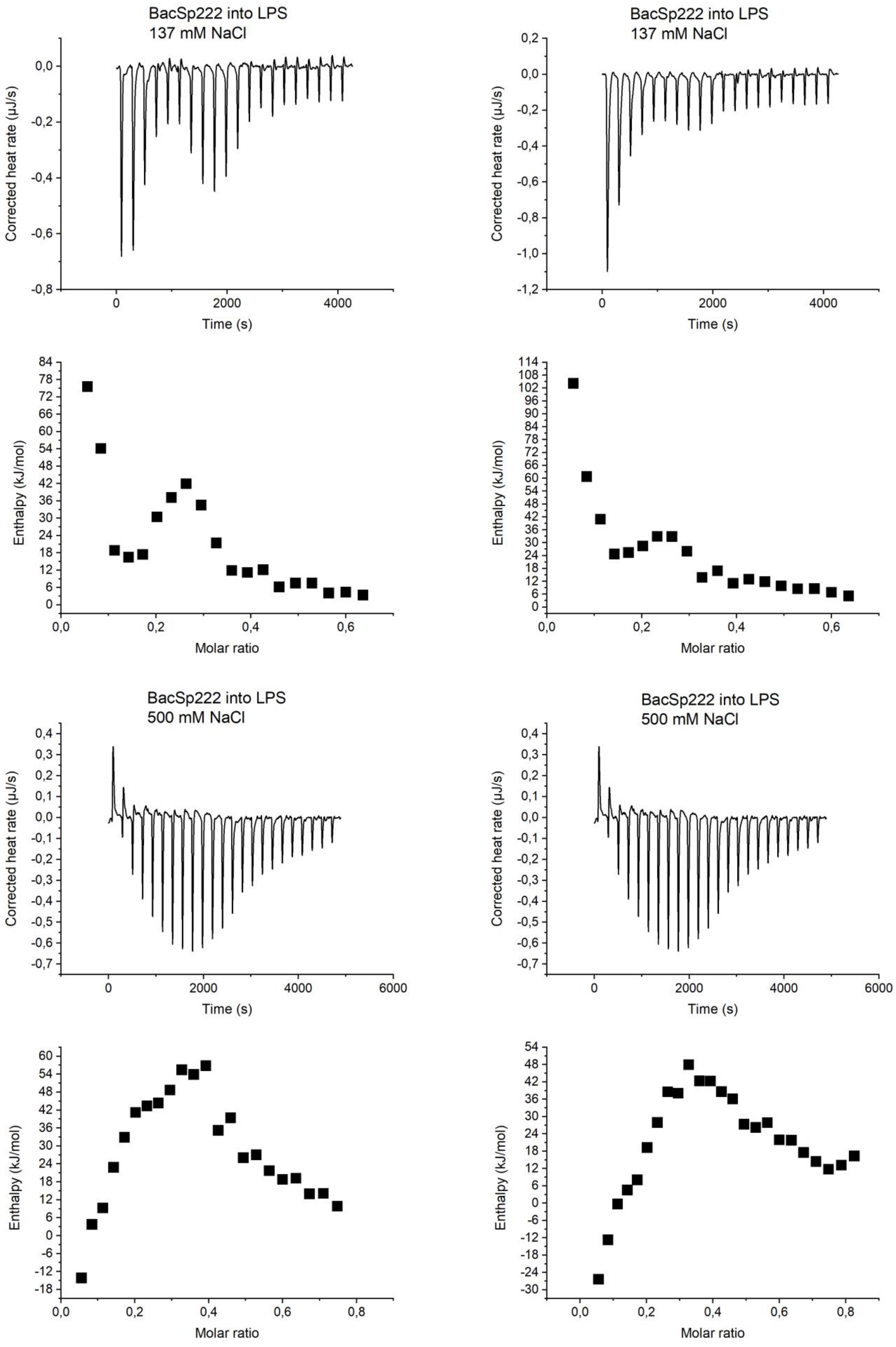

Figure S4. ITC data of BacSp222 interactions with LPS O55:B5 (IEC). Titration was performed at 37 °C by adding 20-25 injections of a 93 µM solution of BacSp222 in a volume of 3 µl to a measuring cell containing 48.5 µM solution of LPS.
